# Post-Myocardial Infarction Left Ventricular Free Wall Rupture Diagnosed by POCUS

**DOI:** 10.24908/pocusj.v11i01.19500

**Published:** 2026-04-22

**Authors:** Jessica Adams, Shannon Overholt, Edward Descallar, Taryn Hoffman

**Affiliations:** HCA Florida Orange Park Hospital, Orange Park, FL, USA

**Keywords:** Ventricular wall rupture, Cardiac POCUS, POCUS, Point of care ultrasound, Emergency medicine, Cardiac tamponade

## Abstract

**Background:**

ST segment elevation myocardial infarction (STEMI) is a common cause of death and disability in the United States. A rare, though highly morbid complication of STEMI is a rupture of the left ventricular free wall. Prompt recognition and action by emergency physicians is essential. This report describes a case of left ventricular free wall rupture in the setting of a STEMI, diagnosed by point of care ultrasound (POCUS) in the emergency department (ED).

**Case Presentation:**

A 72-year-old man presented with severe chest pain and STEMI on his electrocardiogram (ECG), days after an untreated episode of chest pain. Cardiac POCUS revealed a complex pericardial effusion with partially clotted blood, which raised concern for a rupture of the left ventricular free wall.

**Conclusion:**

Cardiac POCUS enabled providers to quickly recognize a complex pericardial effusion and cardiac tamponade in a patient presenting with a STEMI.

## Introduction

The Centers for Disease Control and Prevention (CDC) estimate a total of 750,000 cases of ST elevation myocardial infarction (STEMI) in the United States each year [1]. Of these 750,000 cases, only about 0.01 to 0.5% result in left ventricular free wall rupture, though that number is likely higher as patients with this complication can expire without a known diagnosis. The overall mortality of left ventricular free wall rupture is high. It is responsible for 20% of in-hospital STEMI-related deaths, and can have a mortality of up to 90% if managed conservatively [2]. In this report, we describe a case in which cardiac POCUS was used by emergency physicians to quickly identify the complex pericardial effusion and presumed left ventricular free wall rupture in the setting of recent recurrent STEMI.

## Case Presentation

A 72-year-old man with a medical history only significant for tobacco abuse presented to the emergency department (ED) as a STEMI alert activated by paramedics. The patient had originally developed chest pain about 5 days prior to presentation. He did not seek medical care at that time and his pain resolved the following day. He continued having episodes of pain over the next couple of days until 3 hours prior to presentation, when he developed acute worsening of his symptoms. He described feeling sudden, severe, substernal chest pain that radiated across both of his shoulders. This pain was also associated with nausea but not with dyspnea, leg swelling, or other symptoms. Paramedics performed a 12-lead electrocardiogram (ECG) which showed ST elevations in anterolateral leads. They activated a prehospital STEMI alert and administered 162 mg aspirin during transport. Vitals were stable on their initial assessment, but blood pressure began down trending during transport.

Upon arrival at the ED, the patient had an initial blood pressure of 90/63 mm Hg, a heart rate of 99 beats per minute, and an oxygen saturation on room air of 99%. He appeared to be in moderate distress. Pertinent exam revealed clear lungs, unlabored respirations, benign abdomen, and mild pitting edema of the lower extremities. The patient had delayed capillary refill and weak but symmetric pulses in his extremities.

**Figure 1.**
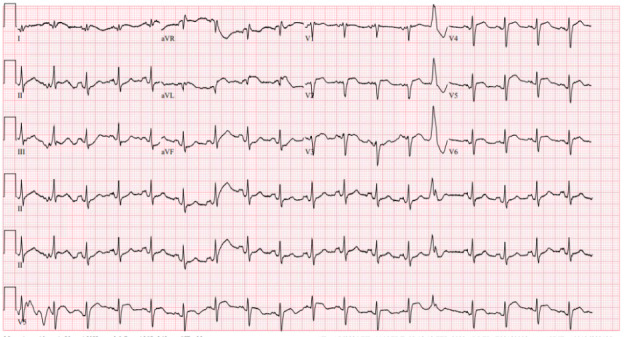
Electrocardiogram (ECG) showing anterolateral ST segment elevation myocardial infarction (STEMI). ST elevations noted in V2-V6 with ST depressions in III and aVF.

**Figure 2.**
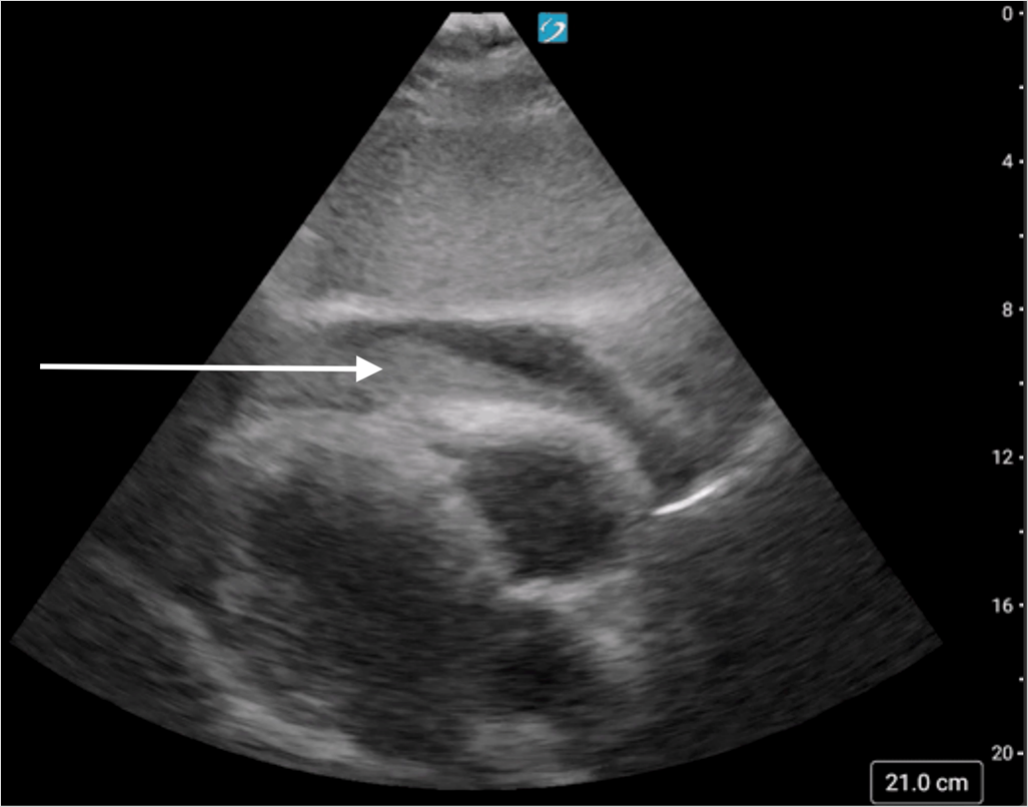
Cardiac point of care ultrasound (POCUS). Subxiphoid view of the heart with large pericardial effusion containing clotted blood (white arrow).

A 12-lead ECG was rapidly performed on arrival (Figure 1). A STEMI alert was reactivated in the ED and the patient was given an additional 162 mg aspirin (for a total of 324 mg), along with an intravenous heparin bolus of 5000 units. ED physicians performed a cardiac POCUS examination (Figure 2, Supplementary Material S1) that revealed a large, mixed-echogenic fluid collection encircling the heart, most consistent with clotted blood. This raised concern for left ventricular free wall rupture, given the history of several days of chest pain. Chest X-ray was performed and read as negative for acute cardiopulmonary abnormality. He started to become more hypotensive, so intravenous fluids were administered and norepinephrine was initiated to maintain adequate blood pressure. Cardiology brought him to the catheterization suite, where he was found to have 60–70% mid-segment stenosis of the left anterior descending artery (LAD) with 100% occlusion of the first diagonal branch. They were unable to pass the wire through the fully occluded vessel, so no stent was placed. Cardiac POCUS in the catheterization lab showed worsening of the pericardial effusion and development of signs of cardiac tamponade. Ultrasound-guided pericardiocentesis was performed which removed 600 cc of blood. This was initially dark red but transitioned to bright red blood. The patient then suffered pulseless electrical activity (PEA) cardiac arrest and massive transfusion protocol was initiated. He was intubated, received multiple doses of epinephrine, 4 units of packed red blood cells, 2 units of fresh frozen platelets, and multiple vasopressors. A pericardial drain was placed. Ultimately, the patient had return of spontaneous circulation (ROSC) after 75 minutes of resuscitation.

Cardiothoracic surgery then took him to the operating room. He went into asystole after draping for surgery, so they initiated cardiopulmonary resuscitation and performed an emergent sternotomy. Upon opening the pericardium, he had 1L of blood with large amounts of clots evacuated, as well as 6.5 L of hemothorax removed. Internal cardiac massage was performed until he was placed on cardiopulmonary bypass. He regained cardiac activity once on bypass, and further repair was done while the heart was beating. The patient was found to have a blowout-type left ventricular free wall rupture with a large anterior tear of infarcted myocardium beginning at the origin of the diagonal arteries. Surgeons repaired the ruptured myocardium with felt strips and suture, but then further rupture occurred so they needed to place an additional patch. Each procedure that was performed yielded further rupture as the myocardium was quite friable. Once hemostasis was achieved at the patch site, the patient was weaned from cardiopulmonary bypass briefly but went back into cardiac arrest and was placed on bypass again. Despite all aggressive measures taken to resuscitate him, the patient's cardiac function was less than 10% and did not improve despite high doses of inotropes and epinephrine. Ultimately, he expired in the operating room.

## Discussion

Left ventricular free wall rupture, though uncommon, is a devastating and often fatal complication of myocardial infarction, most frequently occurring three to five days after the initial infarct. Cardiac POCUS has tremendous benefit when evaluating patients who are at risk for having a left ventricular free wall rupture [3]. As in this case, the use of cardiac POCUS allows an emergency provider to quickly identify an immediate threat to life. The classic finding that is commonly associated with wall rupture is pericardial effusion, from which a patient may rapidly decompensate into cardiac tamponade with right atrial systolic collapse and right ventricular diastolic collapse [3,4]. It is not uncommon to also visualize a papillary muscle rupture in these cases, seen as a hyperechoic structure that prolapses from the left ventricle into the left atrium during systole [5]. As patients with left ventricular free wall rupture are typically unstable, it may be necessary to perform pericardiocentesis to temporarily improve hemodynamics while arranging for emergent surgical intervention for definitive management. Surgical management is the first-line management for this condition. Conservative treatment involves fluid replacement, pressor support, pericardiocentesis as needed, and potentially insertion of an intra-aortic balloon pump, though none of these treatments actually repair the underlying problem [6]. Left ventricular wall rupture is associated with an exceedingly high morbidity and mortality, and early recognition is paramount in improving clinical outcomes and for increasing the chance of a patient's survival.

## References

[1] Fang J, Luncheon C, Ayala C, Odom E, Loustalot F. Awareness of Heart Attack Symptoms and Response Among Adults - United States, 2008, 2014, and 2017. MMWR Morb Mortal Wkly Rep. 2019;68(5):101–106. doi:10.15585/mmwr.mm6805a2 PMC6366680 31851653

[2] Yan L, Wang H, Su B, Fan J, Wang M, Zhao X. Survival after left ventricular free wall rupture following acute myocardial infarction by conservative treatment. Am J Emerg Med. 2021;39:21–23. doi: 10.1016/j.ajem.2020.08.035. 32829991

[3] Lee BW, Cha YS, Hwang SO, Kim YS, Kim SJ. Echocardiographic features of myocardial rupture after acute myocardial infarction on emergency echocardiography. Clin Exp Emerg Med 2023;10(4):393–399. 37280049 10.15441/ceem.23.037 PMC10790066

[4] Alerhand S, Adrian RJ, Long B, Avila J. Pericardial tamponade: A comprehensive emergency medicine and echocardiography review. Am J Emerg Med. 2022;58:159–174. doi: 10.1016/j.ajem.2022.05.001 35696801

[5] Pujari SH, Sharma S, Agasthi P. Left Ventricular Rupture. In: StatPearls. StatPearls Publishing; 2025. Accessed May 26, 2025. http://www.ncbi.nlm.nih.gov/books/NBK559271/

[6] Matteucci M, Fina D, Jiritano F, Meani P, Blankesteijn WM, Raffa GM, Kowaleski M, Heuts S, Beghi C, Maessen J, Lorusso R. Treatment strategies for post-infarction left ventricular free-wall rupture. Eur Heart J Acute Cardiovasc Care. 2019;8(4):379–387. doi: 10.1177/2048872619840876 30932689 PMC6572585

